# Comprehensive Analysis of Severe Viral Infections of Respiratory Tract admitted to PICUs during the Winter Season in Turkey

**DOI:** 10.5005/jp-journals-10071-23177

**Published:** 2019-06

**Authors:** Esra Kockuzu, Benan Bayrakcı, Selman Kesici, Agop Cıtak, Bulent Karapınar, Serhat Emeksiz, Ayşe Berna Anıl, Tanıl Kendirli, Ufuk Yukselmis, Esra Sevketoglu, Şukru Paksu, Onur Kutlu, Hasan Agın, Dincer Yıldızdas, Halil Keskin, Gokhan Kalkan, Arzu Hasanoglu, Mutlu Uysal Yazıcı, Guntulu Sık, Arda Kılınc, Fatih Durak, Oktay Perk, Mey Talip, Nazik Yener, Selcuk Uzuner

**Affiliations:** 1,2,18 Department of Pediatric Intensive Care Unit, Hacettepe University Faculty of Medicine, Ankara, Turkey; 3,19 Clinic of Pediatric Intensive Care Unit, Dr. Sami Ulus Child Health and Disease Training and Research Hospital, Ankara, Turkey; 4 Department of Pediatric Intensive Care Unit, Acıbadem University Faculty of Medicine, Istanbul, Turkey; 5,20,21 Department of Pediatric Intensive Care Unit, Ege University Faculty of Medicine, Izmir, Turkey; 6 Clinic of Pediatric Intensive Care Unit, Ankara Pediatric Hematology Oncology Training and Research Hospital, Ankara, Turkey; 7 Clinic of Pediatric Intensive Care Unit, Ankara Tepecik Training and Research Hospital, Izmir, Turkey; 8,22 Department of Pediatric Intensive Care Unit, Ankara University Faculty of Medicine, Ankara, Turkey; 9 Clinic of Pediatric Intensive Care Unit, Kartal Lutfi Kırdar Education Research Hospital, Istanbul, Turkey; 10,23 Clinic of Pediatric Intensive Care Unit, Bakırkoy Sadi Konuk Education Research Hospital, Istanbul, Turkey; 11,24 Department of Pediatric Intensive Care Unit, Ondokuz Mayıs University, Faculty of Medicine, Samsun, Turkey; 13 Clinic of Pediatric Intensive Care Unit Dr. Behcet Uz Education Research Hospital, Izmir, Turkey; 14 Department of Pediatric Intensive Care Unit, Cukurova University Faculty of Medicine, Adana, Turkey; 15 Department of Pediatric Intensive Care Unit, Ataturk University Faculty of Medicine, Erzurum, Turkey; 16 Department of Pediatric Intensive Care Unit, Gazi University Faculty of Medicine, Ankara, Turkey; 17 Department of Pediatric Intensive Care Unit, Gaziantep University Faculty of Medicine, Gaziantep, Turkey; 12,25 Department of Pediatric Intensive Care Unit, Bezmialem University Faculty of Medicine, Istanbul, Turkey

**Keywords:** Mortality, Multiorgan failure, Pediatric intensive care unit, Seasonal, Viruses

## Abstract

**Objectives:**

To analyze the course of seasonal viral infections of respiratory tract in patients hospitalized in pediatric intensive care units (PICU) of 16 centers in Turkey.

**Materials and methods:**

It is a retrospective, observational, and multicenter study conducted in 16 tertiary PICUs in Turkey includes a total of 302 children with viral cause in the nasal swab which required PICU admission with no interventions.

**Results:**

Median age of patients was 12 months. Respiratory syncytial virus (RSV) was more common in patients over one year of age whereas influenza, human Bocavirus in patients above a year of age was more common (*p* <0.05). Clinical presentations influencing mortality were neurologic symptoms, tachycardia, hypoxia, hypotension, elevated lactate, and acidosis. The critical pH value related with mortality was ≤7.10, and critical PCO_2_ ≥60 mm Hg.

**Conclusion:**

Our findings demonstrate that patients with neurological symptoms, tachycardia, hypoxia, hypotension, acidosis, impaired liver, and renal function at the time of admission exhibit more severe mortal progressions. Presence of acidosis and multiorgan failure was found to be predictor for mortality. Knowledge of clinical presentation and age-related variations among seasonal viruses may give a clue about severe course and prognosis. By presenting the analyzed data of 302 PICU admissions, current study reveals severity of viral respiratory tract infections and release tips for handling them.

**How to cite this article:**

Kockuzu E, Bayrakcı B, Kesici S, Cıtak A, Karapınar K, Emeksiz S, et al. Comprehensive Analysis of Severe Viral Infections of Respiratory Tract admitted to PICUs During the Winter Season in Turkey. Indian J Crit Care Med 2019;23(6):263–269.

## INTRODUCTION

Microbial agents that most commonly lead to childhood infections are viruses. In developed countries, infants and preschool children have viral infection 6–10 times in a year, and school-age children and adolescents have 3–5 times in a year.^1^ In a study published by American Centers for Disease Control and Prevention in 2015, pathogens were detected in 80% of 2,254 pneumonia cases in patients aged between a day and 17 years, 66% of these pathogens were viruses.^2,3^

Viruses may cause several clinical conditions that require hospitalization in a PICU such as bronchiolitis, pneumonia, chronic lung diseases, and more severe manifestations such as myocarditis, encephalitis, and sepsis. Besides the known agents, recently new generation viruses including human metapneumovirus (HMPV), coronavirus, and bocavirus have also been lead to cause these manifestations.^4,5^

In this study, it is aimed to bring out the clinical characteristics of patients who had viral infections requiring PICU admission during winter season in Turkey.

## MATERIALS AND METHODS

PICUs of 16 centers spread to Turkey participated in this retrospective observational study. Children between one month and 18 years of age with a viral cause in the nasal swab, who required PICU admission in the winter 2016–2017 (October–February) period, were included in the study. Approval for this study was obtained from the Hacettepe University Research Ethics Committee. Data form was filled for each patient who had a positive viral polymerase chain reaction in nasal swab sample. The form included demographic data, presentation symptoms, physical examination findings, laboratory findings, isolated virus, indication and diagnosis of PICU admission, radiological findings, underlying disease, organ failure, treatment, accompanying bacterial infection, pediatric mortality scores [Pediatric Risk of Mortality (PRISM), Pediatric Logistic Organ Dysfunction (PELOD)], length of hospital, and PICU stay. Anemia, thrombocytopenia-thrombocytosis, and leukocytosis-leukopenia were recorded according to age group. Oxygen saturation (SO_2_) below 92% is defined as hypoxia. Treatment options were defined as respiratory support treatments, antimicrobial treatments, renal replacement treatments, plasma exchange, and extracorporeal membrane oxygenation (ECMO). Indication for PICU admission was classified as respiratory failure, cardiovascular dysfunction, neurological dysfunction, and multiple organ dysfunction syndrome (MODS). Organ failures were evaluated according to Goldstein 2005 MODS criteria.^6^

Median age of the patients in the study was 12 months. Patients were separated into two groups according to median age. Two groups (0–12 months, 13 months, and over) were compared in terms of presenting findings (clinical, laboratory, and radiological), indication of PICU admission, isolated viruses, organ failures, treatment options, underlying disease, and PICU mortality scores.

Presenting findings, indication of PICU admission, isolated viruses, organ failures, treatment options, underlying diseases, PICU mortality scores, and presence of single or multiple viruses were analyzed through four parameters (duration of MV, PICU, hospital, and mortality).

### Statistical Analysis

Initially descriptive properties of variables (mean, median, number, and percentage) were found. Numerical variables were checked for normal distribution. Student t test was used for normally distributed numerical variables. Mann-Whitney U test was used for variables with no normal distribution when two groups were compared. In comparison with the numerical values of multiple groups, ANOVA was used for variables with normal distribution and Kruskal Vallis test was used for variables with no normal distribution. Comparison of categorical variables was carried out by chi-square test and Fisher Exact test. The most effective presenting finding on mortality was revealed with regression analysis. Multivariate regression analysis was performed for determining mortality risk factor. Critical pH and pCO_2_ value were found with the ROC curve. ROC analysis was performed to evaluate the relationship between blood pH and mortality. The *p*-value <0.05 was considered as significant. The results were evaluated using the Statistical Package for Social Sciences - SPSS 17 (Chicago, USA) program.

## RESULTS

Data of 302 patients with severe viral infections were followed in PICU of 16 centers in Turkey. Out of 302 patients, 113 (37%) were girls and 189 (63%) were boys. Mortality rate was 7.6% (23/302). The mean PRISM score was 37.2±13.2 in patients who died and 11.1±10.3 in patients discharged (*p* <0.001). Median age of patients was 12 (4–36) months. Forty-seven percent of patients were under one year of age and 53% of patients were over one year of age. Mortality rate was 9.2% and 6.3% in each age groups, respectively (*p* = 0.34). It was seen that 45.7% of the patients were admitted to the emergency department, 24.2% were admitted to the inpatient service of the same hospital, and 23.3% of the patients were accepted from the inpatient or emergency services of other hospitals. The mean duration of symptoms before admission to the hospital was 3.9±2.8 days. Duration of stay in PICU was between one day and 116 days, hospital duration was between two days and 120 days.

Symptoms were cough in 78.1%, fever in 62.5%, nasal discharge in 45.6%, gastrointestinal symptoms in 20.8%, neurological symptoms (seizures, changes in consciousness) in 17.5%, and rash in 4.6% of all patients. Hypoxia was found in 49%, tachypnea in 43%, fever in 40%, tachycardia in 34%, and hypotension in 7% of patients. Physical examinations revealed respiratory findings in 86%, prolonged capillary filling in 32%, neurological findings in 14%, and gastrointestinal system findings in 10% of all patients.

Indication of PICU admission was respiratory dysfunction in 71.5%, circulatory dysfunction in 10.2%, neurologic dysfunction in 7.6%, and MODS in 10.5% of patients. At the time of admission and in follow-up, respiratory failure was observed in 91.3%, cardiovascular failure in 22.1%, hematological failure in 15.2%, renal failure in 10.2%, and hepatic failure in 9.9% of patients. In regression analysis, MODS was found as a predictor for mortality [odds ratio: 20.5(95%), confidence interval (CI): 5.8–72.1; *p* = 0.00].

Anemia was found in 45.4%, leukocytosis in 17.8%, leukopenia in 14.2%, thrombocytosis in 19.8%, thrombocytopenia in 14.9%, prolonged INR in 26.6%, and electrolyte imbalance in 74.8% of patients. Acidosis was found in 55.2%, alkalosis in 5.9%, high PCO_2_ in 37.7%, and elevated lactate in 56.9% of patients. The mean lactate value was 6.3±6.1 in patients who died and 2.7±2.5 in patients who were discharged (*p* <0.001). In regression analysis, acidosis was found to be a predictor for mortality (odds ratio 2.5(95%) CI, 1.2–4.8; *p* = 0.007). It was found that pH ≤7.10 negatively affected survival (specificity 97.9% and sensitivity 26.3%). PCO_2_ ≥60 mm Hg was also shown to have negative effect on survival (specificity 78.1% and sensitivity 68.4%). Clinical and laboratory findings at the time of admission of patients associated with mortality are shown in Table 1 and age-related discriminating factors are shown in Table 2.

Anterior-posterior chest radiograph revealed positive findings in 86% of patients (infiltration in 74%, atelectasis in 9%, pleural effusion in 5%, and pneumothorax in 4%). No statistically significant difference was found between the patients who died and discharged in terms of chest radiograph findings.

**Table 1:** Presenting findings associated with mortality

*Presenting Findings*	*Discharged* *% (n)*	*Died* *% (n)*	p*
Tachycardia	33 (92)	56.5 (13)	0.023
Hypotension	6.1 (17)	30.4 (7)	0.001
Cutis marmaratus	6.5 (18)	39.1 (9)	<0.001
Neurologic symptoms	15.8 (44)	39.1 (9)	0.009
Low albumin level	26.2 (73)	65.2 (15)	<0.001
Prolonged INR	23.7 (66)	52.2 (12)	0.003
Hypocalcemia	22.7 (63)	69.6 (16)	0.001
Hypophosphatemia	14 (39)	34.8 (8)	<0.005
Hypomagnesemia	23.7 (52)	47.8 (9)	<0.005
Elevated ALT	15.1 (42)	52.2 (12)	<0.001
Elevated AST	25.7 (71)	60.9 (14)	<0.001
Elevated total bilirubin	12.5 (35)	39.1 (9)	0.001
Elevated direct bilirubin	16.2 (45)	39.1 (9)	0.001
Acidosis	52.7 (147)	87 (20)	0.006

*Number of patients(n); *p is significant when it is <0.05

**Table 2:** Age related discriminating factors

*Presenting Symptoms*	*< 12 months* *% (n)*	*> 12 months* *% (n)*	p*
Respiratory symptoms	79.6(113)	91.9(147)	0.002
Hypoxia	43(61)	55.3(88)	0.032
Fever	72.5(103)	53.8(86)	0.001
Tachycardia	47.2(67)	23.8(38)	<0.001
Neurologic symptoms	22.5(32)	13.1(21)	0.032
Anemia	52.1(74)	39.4(63)	0.026
Leukocytosis	27.9(39)	9.4(15)	<0.001
Thrombocytopenia	24.6(35)	6.3(10)	<0.001
Hyponatremia	46.5(63)	31.3(45)	<0.013
Hypocalcemia	36.9(52)	17.5(27)	<0.001
Hypomagnesemia	33.1(47)	18.8(30)	0.015
High BUN level	49.3(70)	20.6(33)	<0.001
High creatine level	21.8(31)	13.1(21)	0.045
Hypoalbuminemia	38.7(55)	20.6(33)	0.001
Indication of Picu Admission			
Pulmonary dysfunction	59.2(84)	82.5(132)	<0.001
Neurological dysfunction	13.4(19)	2.5(4)	<0.001
Circulatory dysfunction	14.1(20)	6.9(11)	<0.039
Organ Failure during Hospitalization and Follow-Up			
Neurologic failure	21.8(31)	8.8(14)	0.005
Hematologic failure	21.9(30)	10.1(16)	0.005
Isolated Virus			
RSV	23.2(33)	55(88)	<0.001
Influenza	27.5(39)	13.8(22)	0.003
Human bocavirus	12.7(18)	3.8(6)	0.004
Others			
Need for mechanical ventilator	37.5(60)	54.2(77)	0.004
Hospitalization duration *	12 (9-23) days	17 (9-28) days	0.04
Underlying disease	65.5(93)	34.5(68)	<0.001

*Number of patients (n); *25–75% and median values of hospitalization duration are given; *p is significant when it is <0.05

**Table 3:** Relationship of virus type with mortality

*Viruses*	*(A)* *% (n)*	*(B)* *% (n)*	*(C)* *% (n)*	*(D)* *% (n)*	*(E)* *(D/C) %*
RSV	40 (121)	17.4 (4)	40.6 (106)	21.1 (4)	3.7
Influenza	20.1 (61)	17.4 (4)	17.2 (45)	21.1 (4)	8.8
Rhinovirus	19.2 (58)	17.4 (4)	14.9 (39)	5.3 (1)	2.5
Parainfluenza	12.9 (39)	26.1 (6)	11.1 (29)	21.1 (4)	13.7
Human bocavirus	7.9 (24)	17.4 (4)	4.5 (12)	5.3 (1)	8.3
HMPV	5.2 (16)	4.3 (1)	5.3 (14)	5.3 (1)	7.1
Adenovirus	4.9 (15)	4.3 (1)	3 (8)	5.3 (1)	12.5
Coronavirus	3.9 (12)	13 (3)	3 (8)	15.8 (3)	37.5

A, number of viruses (multiple reproduction included); B, isolated viruses in patients who died (multiple reproductions included); C, number of viruses (multiple reproduction excluded); D, isolated viruses in patients who died (multiple reproduction excluded); E, rate of mortality for a single virus (D/C); Number of patients (n)

**Table 4:** Relationship between indication of PICU Admission, organ failure, and virus type (%)

*Indications of hospitalization and organ failures*	*RSV*	*İnfluenza*	*Rhinovirus*	*Parainfluenza*	*Human bocavirus*	*Human metapneu-movirus*	*Corona-virus*	*Adeno-virus*	*Multiple viruses*	p*
Circulatory dysfunction	9.4	11.1	2.6	13.8	8.3	7.1	12.5	37.5	12.2	0.43
Pulmonary dysfunction	83	37.8	76.9	65.5	50	92.9	75	37.5	82.9	0.72
Neurologic dysfunction	4.7	26.7	7.7	6.9	8.3	0	0	0	0	0.058
Multiorgan failure	2.8	24.4	10.3	13.8	33.3	0	12.5	25	7.3	0.48
Respiratory failure	94.3	75.6	92.3	96.6	83.3	100	100	87.5	95.1	0.74
Cardiovascular failure	15.2	33.3	12.8	27.6	41.7	21.4	25	62.5	19.5	0.34
Neurologic failure	5.7	37.8	17.9	24.1	25	0	12.5	0	9.3	0.37
Hepatic failure	3.8	20	10.3	10.3	16.7	0	37.5	25	7.3	0.28
Renal failure	2.8	24.4	7.7	13.8	25	0	25	25	7.5	0.41
Hematologic failure	8.7	31.1	13.9	10.3	16.7	7.7	37.5	50	12.5	0.18

*p is significant when it is <0.05

Out of all, 53.3% patients had underlying disease [neurologic in 17.8%, respiratory in 16.5%, cardiovascular in 12.5%, immunological in 6.2%, hematological-oncological in 5.9%, renal in 2.3%, and others (metabolic disease, chromosomal anomalies) 9.5%]. Sixty percent of patients who died had underlying disease. This rate was 52% in discharged patients (*p* >0.05).

The total number of viruses detected in nasal swabs of patients hospitalized in PICU, number of viruses when multiple reproductions were included and excluded in the patients who died, mortality rates when the viruses were evaluated in themselves are shown in Table 3. Multiple viruses were detected in 13% of all patients. Multiple viruses isolated was in 17.3% of the patients who died and in 13.2% of the discharged patients (*p* >0.05). For patients detected with a single-virus infection, there was statistically no difference between the species of the virus, between patients who survived, and patients who died (*p* = 0.195). When the viruses were evaluated among themselves and patients with multiple viruses were excluded, 37% of patients with coronavirus were found to have died (Table 3).

Median age of patients with influenza was 41 (11–75) months and median age of patients with the viruses other than influenza was 10 (3.5–28) months (*p* <0.001). The median PRISM score was 22 (8–27) and median PELOD score was 11 (3–21) in patients with influenza. The median PRISM score was 9 (3–17) and median PELOD score was 10 (1–15) in patients with the viruses other than influenza (*p* <0.005). The relationships between the indication of PICU admission, organ failures at the time of admission or revealed at follow-up, and species of viruses isolated are shown in Table 4. Among all, 60.2% of patients received antiviral therapy, 94.3% antibiotic therapy, 15.8% antifungal therapy. 47.4% of patients who died, and 61.6% of the patients discharged received antiviral therapy (*p* = 0.22). The median duration of stay on MV was 4 (2–5) days, median duration of stay in PICU was 7 (4–15), and median duration of hospital was 15 (10–27) days in patients who received antiviral therapy. The median duration of stay on MV was 3 (2–5) days, median duration of stay in PICU was 7 (3–13), and median duration of hospital was 15 (8–28) days in the patients who did not receive antiviral therapy (*p* >0.05). It was found that virus species did not affect mortality between patients who received and did not receive antiviral therapy (Table 5).

**Table 5:** Effect of antiviral treatment on mortality according to virus species

*Virus species*	*Antiviral treatment*				p*
	*Without oseltamivir*		*Oseltamivir*		
	*Patient number*	*%*	*Patient number*	*%*	
Parainfluenza	22	18,5	7	3,8	0.182
RSV	43	36,1	63	34,4	
İnfluenza	6	5,0	39	21,3	
Rhinovirus	18	15,1	21	11,5	
HMPV	4	3,4	10	5,5	
Human bocavirus	5	4,2	7	3,8	
Coronavirus	3	2,5	5	2,7	
Adenovirus	2	1,7	6	3,3	
Multiple viruses	16	13,4	25	13,7	

*p is significant when it is <0.05

Bacterial infection was detected in the cultures of 16% of patients. There was bacterial infection in 43.5% of patients who died and in 14.5% of patients who were discharged (*p* = 0.001). Secondary bacterial infection was detected in 37.5% of patients with coronavirus, 25% of patients with adenovirus, 20% of patients with influenza, 17,9% of patients with rhinovirus, 17.2% of patients with parainfluenza, 14.3% of patients with RSV, and 7.1% of patients with HMPV. There was statistically no difference between the viruses in terms of development of secondary bacterial infection (*p* = 0.5).

Of all, 92.3% of patients required respiratory support treatments. Renal replacement therapy was performed in 6.6%, plasma exchange in 5.2%, and ECMO in 1.9% of patients.

## DISCUSSION

Viral respiratory tract infections are the most common infections worldwide. They are the most important cause of mortality in all age groups, particularly in children.^7^ This retrospective observational study was conducted in order to analyze the course of seasonal viral infections of respiratory tract in patients hospitalized in PICUs of 16 centers representing the whole country. There are a bunch of studies in literature investigating the relationship between viruses and morbidity, comparison of single-multiple viruses or relationship between diseases and viruses.^8–18^ Current study is multi-directional and has comprehensively analyzed all these issues in a very large population requiring PICU.

Viral respiratory tract infections constitute significant proportion of patients admitted to PICUs in winter, and these patients require advanced intensive care support with high mortality and morbidity rates. In line with this, our study group shows a very high mortality rate as 7.6%, which supports the justification of this multicenter survey. Although respiratory tract viruses commonly cause lower respiratory tract infections, presentation with extrapulmonary clinical manifestations are also likely in children under one year of age.^8,19^ Similarly, the most common cause of hospitalization was respiratory symptoms in our study. Fever, neurological symptoms, circulatory disorders, and shock were more common indications for hospitalization in patients under one year compared to older children (Table 2). In patients over one year of age presenting with respiratory symptoms, hypoxia as well as a need for MV were more common. We attributed this situation to RSV, which was more frequently observed in this age group. In the literature, RSV is more prevalent in infancy for PICU admission though influenza and bocavirus that was more common in our patients less than one year of age.^1,9,10^

We statistically evaluated all initial symptoms (clinical, laboratory, and radiological) and virus species through four parameters (duration of stay on mechanical ventilation (MV), duration of stay in PICU, duration of hospitalization, and mortality). Accordingly, we assessed relationship among presenting symptoms, virus species, and severity of disease. It was observed that presenting symptoms that have an effect on mortality were neurologic symptoms, hypotension, tachycardia, and hypoxia. It was also found that liver and renal function test abnormalities, higher lactate values were related with death. Eighty-seven percent of mortalities are presented with acidosis (Table 1). The critical pH value related with mortality and critical PCO_2_ was ≤7.10 and ≥60 mm Hg, respectively. In regression analysis, acidosis alone was found to increase mortality by 2.5 folds. In the light of these findings, it can be predicted that patients with neurological symptoms, tachycardia, hypotension, low levels of pulse sO_2_, acidosis, and impaired liver or renal function at the time of admission will have a higher morbidity and mortality.

Kumar et al. showed that organ failure in patients hospitalized in ICU with H1N1 infection increased mortality.^11^ Also, Kendirli et al. demonstrated the correlation between organ failures and mortality in the patients who presented with H1N1 infection.^12^ Similarly, in our study, mortality rates were higher and in regression analysis, we demonstrated that initial presentation with MODS increased mortality by 20-folds.

In a study, it was shown that RSV was the most common agent in patients who needed intensive care.^20^ This is definitely compatible with our findings.

Spaeder and Fackler reported that virus species did not affect durations of stay in PICU and hospital, as well as development of MODS and mortality.^21^ In our study, we also investigated virus species organ failure relationship and emerging indications for PICU admission. We demonstrated that there was no correlation between virus species and diagnosis of admission to PICU or type of organ failure developed (Table 4). However, 63% of the patients who died in 2017 winter season had RSV, parainfluenza or influenza. Secondary bacterial agent was observed in about half of the patients who died. It is not easy to blame viral agents to be the leading causes of mortality because of the frequent accompaniment of secondary bacterial agents. Similar to the literature, we found that secondary bacterial infections negatively affected mortality.^15,22,23^

In the present study, multiple viruses were detected in 13% of the patients. Although there are also contrary opinions,^24–26^ detection of multiple viruses does not affect mortality and duration of stay in PICU.^22,27,28^ Similarly, we showed that multiple viruses did not affect mortality.

When multiple virus isolated patients were excluded, coronavirus revealed to be the most lethal virus as three out of eight patients with coronavirus died (Table 3). Coronavirus is known as the primary agent of upper respiratory tract infection and common cold.^29^ Coronavirus had been defined as the causal agent of SARS (Severe Acute Respiratory Syndrome), which more severely progresses in adults, while the disease lasts shorter and shows milder progression in children.^30^ Unlike the literature, coronavirus presented severe symptoms with organ failures in our group of patients (Table 4). Lower respiratory tract infections caused by coronavirus types out of SARS are rare and some studies found the incidence of pneumonia and bronchitis as <5%.^31–36^ Therefore, in our study, the incidence of coronavirus was low that is consistent with the literature, but unlike that coronavirus caused lower respiratory tract infections and emerged as the most lethal viral agent (Table 3). However, 36% of the patients with coronavirus had secondary bacterial infections; so, coronavirus is not solely responsible for mortality.

Although secondary infections were found in only 16% of all patients, we found that 94% of the patients who presented with viral infections received antibiotics.

Sixty-two percent of the patients received antiviral treatment (oseltamivir) because antiviral therapy was started before nasal swab results in patients with severe infection findings. We found that antiviral therapy did not affect mortality (Table 5) including the influenza subgroup. Though, there are studies in literature showing that oseltamivir initiated in the early period was effective.^37–39^ However, weakness of our study is that the data we gathered is not capable of differentiating the initiation timing for antiviral therapy. Further studies are needed for this issue. Influenza is already known as the only agent that benefits from oseltamivir. When we separately evaluated the influenza subgroup, we found the median patient age as 41 months and higher PRISM and PELOD scores compared with rest of the viral agents. Influenza was more common in older children and progressed more severely. Therefore, oseltamivir can be preferred in children especially over one year of age and who presented with more severe symptoms because they are more likely to have influenza.

Consistent with the literature, comorbidities were common in the present study.^9,12–14,20^ Although 60% of the patients who died had an underlying disease, a statistical correlation is lacking as described in the literature.^9,12^

In our study, it was found that mechanical respiratory support was administered to the majority of the patients and renal replacement therapy was applied in the group, which had organ failure and progression. As expected, we found that PRISM and PELOD scores were higher in patients who died.

## CONCLUSION

We found that tachycardia, hypotension, hypoxia, acidosis, impaired liver or renal tests, and MODS at the time of admission are the factors associated with mortality and predictors of a severely progressing disease. It was found that acidosis increased mortality by 2.5-folds and MODS by 20-folds. Respiratory symptoms were common and besides respiratory symptoms, incidence of circulatory disorders and shock findings were also high in patients less than one year of age. Fever, tachycardia, neurologic symptoms, the incidence of anemia, leukocytosis, thrombocytopenia, impaired renal tests, and electrolyte imbalance were high in patients less than one year of age. Consequently, acidosis and MODS at the time of admission or a patient less than one year of age with symptoms mentioned above should alert clinicians for a possible poor prognosis. Although we could not show the relationship of virus type with severity and mortality, RSV, parainfluenza, and influenza were the commonly isolated viruses in the patients who died. Coronavirus was found as the most lethal virus. Although coronavirus is known as the primary agent of upper respiratory tract infection and common cold, this result of our study can be attributed to the incidence of secondary bacterial infections being high in the patients with coronavirus detected. With this context, further studies are needed to investigate the relationship between coronavirus and mortality.

In our study, we could not demonstrate effects of oseltamivir on mortality, duration of hospitalization, and stay in the PICU including influenza subgroup but there are studies reporting that oseltamivir initiated within first 48 hours is beneficial. Therefore, oseltamivir can be preferred in children who present with more severe symptoms because they are more likely to have influenza.
